# Impacts on product quality attributes of monoclonal antibodies produced in CHO cell bioreactor cultures during intentional mycoplasma contamination events

**DOI:** 10.1002/bit.27436

**Published:** 2020-06-04

**Authors:** Erica J. Fratz‐Berilla, Phillip Angart, Ryan J. Graham, David N. Powers, Adil Mohammad, Casey Kohnhorst, Talia Faison, Sai Rashmika Velugula‐Yellela, Nicholas Trunfio, Cyrus Agarabi

**Affiliations:** ^1^ Division of Biotechnology Review and Research II, U.S. Food and Drug Administration Center for Drug Evaluation and Research, Office of Pharmaceutical Quality, Office of Biotechnology Products Silver Spring Maryland; ^2^ Division of Product Quality Research, U.S. Food and Drug Administration Center for Drug Evaluation and Research, Office of Pharmaceutical Quality, Office of Testing and Research Silver Spring Maryland; ^3^ Department of Chemical Engineering University of Massachusetts Lowell Lowell Massachusetts; ^4^ Nova Biomedical Waltham Massachusetts; ^5^ Sartorius Stedim Data Analytics Bohemia New York

**Keywords:** biomanufacturing, bioprocessing, Chinese hamster ovary (CHO) cell culture, critical quality attribute (CQA), monoclonal antibody (mAb), mycoplasma, partial least squares (PLS) regression, principal component analysis (PCA)

## Abstract

A mycoplasma contamination event in a biomanufacturing facility can result in costly cleanups and potential drug shortages. Mycoplasma may survive in mammalian cell cultures with only subtle changes to the culture and penetrate the standard 0.2‐µm filters used in the clarification of harvested cell culture fluid. Previously, we reported a study regarding the ability of *Mycoplasma arginini* to persist in a single‐use, perfusion rocking bioreactor system containing a Chinese hamster ovary (CHO) DG44 cell line expressing a model monoclonal immunoglobulin G 1 (IgG1) antibody. Our previous work showed that *M. arginini* affects CHO cell growth profile, viability, nutrient consumption, oxygen use, and waste production at varying timepoints after *M. arginini* introduction to the culture. Careful evaluation of certain identified process parameters over time may be used to indicate mycoplasma contamination in CHO cell cultures in a bioreactor before detection from a traditional method. In this report, we studied the changes in the IgG1 product quality produced by CHO cells considered to be induced by the *M. arginini* contamination events. We observed changes in critical quality attributes correlated with the duration of contamination, including increased acidic charge variants and high mannose species, which were further modeled using principal component analysis to explore the relationships among *M. arginini* contamination, CHO cell growth and metabolites, and IgG1 product quality attributes. Finally, partial least square models using NIR spectral data were used to establish predictions of high levels (≥10^4^ colony‐forming unit [CFU/ml]) of *M. arginini* contamination, but prediction of levels below 10^4^ CFU/ml were not reliable. Contamination of CHO cells with *M. arginini* resulted in significant reduction of antibody product quality, highlighting the importance of rapid microbiological testing and mycoplasma testing during particularly long upstream bioprocesses to ensure product safety and quality.

## Graphical abstract

A mycoplasma contamination event in a biomanufacturing facility can result in costly cleanups and potential drug shortages. The authors investigated the effects of mycoplasma presence on the critical quality attributes of a monoclonal IgG1 produced in CHO cells and found that contamination increased acidic charge variants, increased DNA contamination, and led to very high percentages of high mannose species with 6 or more mannose residues.

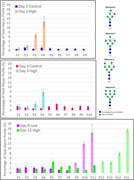

## 1 INTRODUCTION

Mycoplasmas are small (0.1‐0.3 µm diameter) bacteria without cell walls, allowing for penetration of the typical 0.1‐0.22 µm sterilizing‐grade filters used in scientific research and biomanufacturing (Razin, 2006). Mycoplasma contamination risk in biomanufacturing is lower than the risk in academic research laboratories where it is still commonplace to use media supplemented with animal‐derived serum components (Drexler & Uphoff, 2002), but even with the use of chemically derived media as the norm (Fletcher & Harris, 2016), contamination risk is still present via other medium additives and manual manipulations of cell lines. Mycoplasma incidence rates are estimated to be 15–35% in banked cell lines and 0.44–6.70% within the biopharmaceutical industry, depending on the detection assay used (Armstrong, Mariano, & Lundin, 2010; Chandler, Volokhov, & Chizhikov, 2011; Drexler & Uphoff, 2002). Of these contamination events, 95% of the mycoplasma species identified were one of the following: *Mycoplasma orale*, *Mycoplasma arginini*, *Mycoplasma hyorhinis*, *Mycoplasma fermentans*, *Mycoplasma hominis*, or *Acholeplasma laidlawii* (Drexler & Uphoff, 2002; Kljavin, 2007). Despite comprehensive control strategies (Guilfoyle et al., 2013; Quality Risk Management Q9, 2005; Rosenberg et al., 2011), mycoplasma can potentially contaminate a bioprocessing scheme through other cell culture medium components (Drexler & Uphoff, 2002; Kljavin, 2007, 2011; Windsor, Windsor, & Noordergraaf, 2010) or during manual manipulation of cell lines such as cell banking and cell line development (Nikfarjam & Farzaneh, 2012). Some species of mycoplasma such as *Acholeplasma laidlawii* (Windsor et al., 2010) cause occult contaminations of cell cultures with minimal visible changes to cell health or cell culture performance, while other mycoplasma contaminations can subtly alter the culture performance through potential mechanisms including competition for culture nutrients and alteration of the host cell expression profile (Rottem, 2003). Although there are differences among species, mycoplasma tends to grow significantly slower than other bacteria, with doubling times ranging anywhere from 1 to 9 hr (Drexler & Uphoff, 2002). The small size, slow growth, and sometimes subtle effects of mycoplasma make these bacteria particularly ideal for adopting early detection strategies in biomanufacturing.

The ability of mycoplasma to evade detection and grow in mammalian cell cultures creates risk in some ways more like viral contamination than typical bacterial contamination. Mycoplasma contamination, much like viral contamination in the biotechnology industry, although rare, can lead to millions of dollars spent for investigation and decontamination measures, drug shortages, and damage to the public's confidence in the manufacturer (Barone et al., 2020). Usually when biopharmaceutical manufacturers find mycoplasma in their cultures, they immediately decontaminate after taking a limited number of culture samples for specification and test raw material samples to trace the source of the contamination (Angart, Kohnhorst, Chiang, & Arden, 2018). To understand how a mycoplasma contamination event may appear in a typical biomanufacturing scheme and provide knowledge on how process monitoring may identify a mycoplasma contamination event in the manufacturing environment, our group developed models of early‐stage process and late‐stage process bioreactor culture contamination events using *Mycoplasma arginini* (*M. arginini*) and Chinese hamster ovary (CHO) cells grown in serum‐free medium expressing a model immunoglobulin 1 (IgG1) product. We examined the growth kinetics of *M. arginini*, a species selected due to its perceptible effects on CHO cells in pilot coculture shake flask studies (Faison et al., 2019; Wang et al., 2017), in a controlled bioreactor environment and the effects on CHO cell culture performance and process parameters (Fratz‐Berilla et al., 2019). We investigated the effects of mycoplasma presence on CHO cell health, productivity, culture metabolism, and process parameters compared to uninfected controls and observed effects 2–6 days after *M. arginini* introduction to the culture, depending on the viable cell density and perfusion conditions at the time of contamination (Fratz‐Berilla et al., 2019). In this report, we investigate the changes in IgG1 product quality produced by CHO cells induced by the *M. arginini* contamination events. We observed changes in charge variant profiles, purity, and glycan patterns that were further modeled using principal component analysis (PCA) to explore the relationships among *M. arginini* contamination, CHO cell growth and metabolites, and IgG1 product quality attributes. Finally, partial least square (PLS) models using NIR spectra collected every 2 min from the bioreactor cultures were used to establish predictions of high levels (>10^4^ colony‐forming unit [CFU/ml]) of *M. arginini* contamination.

## 2 MATERIALS AND METHODS

### 2.1 Rocking perfusion bioreactor operation and mycoplasma spiking

Seed train expansion and bioreactor operation were performed as previously described (Fratz‐Berilla et al., 2019). Briefly, we inoculated a recombinant CHO DG44 cell line that expresses a model chimeric IgG1 in a ReadyToProcess WAVE™ 25 rocker (GE, 28988000) operated in dual mode with two 2‐L single‐use cell bags (GE, CB0002L10‐3; 1 L maximum operating volume) containing porous polyethylene‐based perfusion filters. *M. arginini* was spiked into bioreactors either early in culture, before the start of perfusion when CHO cells were ~2 × 10^6^ cells/ml, or late in culture, during perfusion of 2 L/day when CHO cells were 9–12 × 10^6^ cells/ml and compared to control uncontaminated bioreactors (Fratz‐Berilla et al., 2019). The mycoplasma spike for the bioreactor was prepared from a frozen stock of *M. arginini* in 45% glycerol thawed at room temperature in a laminar flow hood using a sterile syringe and disposable pipette basin. The mycoplasma stock was mixed with 3 ml media (OptiCHO/l‐Glutamine + soy hydrolysate) warmed to 37°C to seed a final target concentration of 10^1^ (low spike) or 10^2^–10^3^ (high spike) CFU/ml in 1 L bioreactor volume. A negative control containing an identical volume of media and supplements only was also prepared for the uncontaminated bioreactor and control bioreactor cultures were confirmed to be uncontaminated during the entirety of the run. Each reactor was connected to a flow cell of a near‐infrared (NIR) spectrometer (Sartorius Stedim) via the perfusion filter line in which culture material was continuously pumped either back into the bioreactor (batch mode) or into the perfusate collection carboy (perfusion mode). Three 14–19 day runs of two bioreactor cell bags, as described in Table 1, were completed.

**Table 1:** Description of bioreactor runs

Bioreactor name	Run length (days)	Length of perfusion (days)	Day of perfusion start	Day of mycoplasma spike	Mycoplasma spike level (CFU/ml)	Description
Day 2‐High	14	4^a^	5	2	220	Model for early‐stage high‐level bioreactor contamination before perfusion start
Day 2‐Control	14	9	5	–	–	Control bioreactor (uncontaminated)
Day 3‐High	14	4^a^	4	3	260	Model for early‐stage high‐level bioreactor contamination before perfusion start
Day 3‐Control	14	10	4	–	–	Control bioreactor (uncontaminated)
Day 9‐Low	19	11	5	9	15	Model for late‐stage low‐level bioreactor contamination during perfusion operation
Day 12‐High	19	15	5	12	300	Model for late‐stage high‐level bioreactor contamination during perfusion operation

Abbreviation: CFU, colony‐forming unit.

^a^ Perfusion of the bioreactor was stopped when CHO cell viability fell below 50%.

### 2.2 Amino acid characterization by liquid chromatography–mass spectrometry

The crude bioreactor medium was centrifuged and passed through a 0.22‐μm polyethersulfone filter (MilliporeSigma, SLGP033RS). A perchloric acid cleanup was used to remove protein and particulate matter, which involved mixing filtered bioreactor medium with 0.4N HClO_4_ (Sigma‐Aldrich, 311421) at a 1:1 ratio and centrifuging at 1,962*g* for 5 min at RT. The clarified medium was collected to be analyzed by liquid chromatography–mass spectrometry (LC‐MS).

A Q‐ToF mass spectrometer (Waters Xevo G2, 720005074EN; run in ESI positive sensitivity mode) coupled to a UPLC I‐Class (Waters ACQUITY, 720003920EN) was used for analysis. We used an Intrada Amino Acid column (Imtakt, WAA24) to perform normal phase chromatography and separate the amino acids. The buffers used were (a) acetonitrile (Fisher Chemical, A955‐500) + 0.1% formic acid (Fisher Chemical, A117‐50); and (b) 100 mM ammonium formate (Sigma‐Aldrich, 70221–25G‐F), with a flow rate of 0.6 ml/min, a gradient time of 15 min, and column temperature of 40°C. Amino Acid Standards (Agilent, 5061‐3330) were utilized to generate a calibration curve (20–2700 pmol/µl) in the QuanLynx software (Waters, WA20366), which was used to calculate the concentrations of amino acids detected in the prepared bioreactor medium samples. Medium samples were run in triplicate, with error bars indicating standard deviations. Additional information on this method can be found in past work (Velugula‐Yellela et al., 2019).

### 2.3 Antibody purification

The perfusate obtained each day was purified using two AKTA Avant Purification systems (GE Healthcare Life Sciences, 28930842), one for uncontaminated culture fluid and one for mycoplasma‐contaminated culture fluid, as previously described (Velugula‐Yellela et al., 2019). The stationary phase was Prosep® vA Ultra resin (Millipore, 113115830) packed to 10 cm in a HiScale 16/20 glass housing (GE Healthcare Life Sciences, 28964441) with 16 mm inner diameter. The resulting purified protein solution was neutralized using 1 M Tris Base (Thermo Fisher Scientific, BP154‐1) to a final pH 5–6. After neutralization, purified protein was concentrated with a 4‐ml Amicon 50 kDa cutoff filter (Millipore, Z740177‐24EA) to a final concentration of ~6 mg/ml. To adjust the protein concentration for size and charge variant assays, samples were diluted in 0.1 M acetic acid (Thermo Fisher Scientific, A38S‐500) buffered to pH 5.56 with 1 M Tris base, hereafter referred to as Tris‐acetate buffer. Protein concentrations were measured using Nanodrop™ 2000/200c spectrophotometer (Thermo Fisher Scientific, ND‐2000C) and the Nanodrop's default extinction coefficient of 1.37 ml/(mg × cm) for mAb samples. Absorbance ratios (260:280 nm) were also recorded for each sample.

### 2.4 Protein aggregation (size exchange chromotography multiangle light scattering)

The aggregate analysis was performed on a UPLC (Agilent 1290 Infinity I, G7120A) connected to a Multiangle Light scattering detector (Wyatt μDawn) and refractive index detector (Optilab UT‐rEX). Size exclusion chromatography was performed with a TSKgel UP‐SW3000 4.6 mm ID × 30 cm/L (Tosoh Biosciences, 003449) and 1 × PBS (Corning, 46‐013‐CM) as the mobile phase operating at a flow rate of 0.4 ml/min. IgG1 samples were diluted to a concentration of 2 mg/ml in Tris‐acetate buffer and 5 μl of the sample was injected per run. Peak quantitation was performed in Astra using the unit variance (UV) absorbance signal from the Agilent DAD detector at 280 nm and average molecular weight determinations were calculated for the same peak areas, using a refractive index increment (dn/dc) of 0.185.

### 2.5 Purity and fragmentation (micro‐capillary electrophoresis‐sodium dodecyl sulfate)

Reduced and nonreduced size analysis was performed with a LabChip® GXII HT Touch™ (Perkin Elmer, CLS138160), utilizing the Protein Express chip (Perkin Elmer, 760499) and Protein QC Reagent Kit (Perkin Elmer, CLS960014). Concentrated samples were diluted to 1 mg/ml and then analyzed according to the manufacturer's protocol. Briefly, 2.5 μl of diluted protein was mixed with 18 μl of sample buffer with 8.75 mM Iodoacetamide (Thermo Fisher Scientific, 90034) or 35 mM DTT (Sigma‐Aldrich, D0632‐1G) for nonreducing or reducing conditions, respectively. Samples were then heated to 70°C for 10 min, cooled to room temperature and mixed with 60 μl of water. The analysis was performed in triplicate with the Protein QC assay after calibration with the VeriMab standard.

Electropherograms were exported to Empower 3 FR5 and analyzed peak areas determined after smoothing the data with a means movement filter (filter width: 15) and peaks called using the Gaussian skim and shoulder detection features. Peak sizes were determined in GXII Reviewer using the internal reference ladder (Protein QC Reagent Kit, CLS960014). Overestimates in the A280 reading were determined by taking samples normalized to 1 mg/ml based on their A280 reading and comparing the peak areas on nonreduced micro‐capillary electrophoresis‐sodium dodecyl sulfate (µCE‐SDS) of low nucleic acid samples (280/260 < 0.52) with the remaining samples.

### 2.6 Charge variant analysis

The relative mAb charge variant distribution was measured with the LabChip® GXII HT Touch™ (Perkin Elmer, CLS138160), the DNA 5K/RNA/CZE Chip (Perkin Elmer, 760435), and the Protein Charge Variant Reagent Kit (Perkin Elmer, CLS760670), according to the manufactures’ protocol. Briefly, purified IgG1 was diluted to 3.3 mg/ml in Tris/acetate buffer and then 80 μl was desalted with a 75 µl 7 K MWCO Zeba desalting column (Thermo Fisher Scientific, 89878). The concentration of each sample was measured using its A280 and then for each labeling reaction, adjusted to 25 μl of IgG1 at 2 mg/ml concentration with water. Labeling was performed as specified, using N,N‐dimethylformamide (Acros Organics, 61094‐1000) in addition to the components provided in the charge variant labeling kit. Care was taken to mix each sample in‐between the addition of each reagent. The DNA 5K/RNA/CZE Chip was prepared with pH 7.2 reagents and samples were analyzed with a 90 s analysis time. All samples were analyzed in triplicate. Peak areas were determined using Empower 3 FR5, similar to the analysis of µCE‐SDS described above.

### 2.7 Glycan characterization

This procedure is demonstrated in the *Journal of Visualized Experiments* (Velugula‐Yellela et al., 2019). Briefly, the glycans were cleaved from the denatured, purified mAb using PNGase F and labeled using RapiFlour‐MS (Waters, 176003712). The labeled glycans were isolated using HILIC chromatography (Waters, 176003712). Glycan identities were established using glucose units from the RapiFlour‐MS Dextran Calibration Ladder (Waters, 186007982), with mass spectrometry used to validate identifications. Three technical replicates were run for each sample.

### 2.8 Metals analysis (inductively coupled plasma mass spectrometry)

Quantitation of iron, copper, zinc, and manganese in daily perfusate samples was performed using an inductively coupled plasma mass spectrometry instrument (Perkin Elmer, NexIon 300D) as described previously (Mohammad et al., 2019).

### 2.9 Data preprocessing

Process data, including nutrient and mycoplasma concentrations, were compiled alongside glycosylation, charge variant, and aggregation quality parameters for both Day 9‐Low and Day 12‐High, respectively. All process and product quality data before the first perfusate were excluded so that only data from the first perfusate through harvest were considered. All datasets were imported into SIMCA 13.0 (Sartorius Stedim) and mean‐centered and mean‐scaled by UV before multivariate analysis. NIR spectra from each run were filtered by 15‐min intervals and assigned to corresponding mycoplasma concentrations as rounded to the nearest minute. Spectra were then treated with a series of common NIR filtration techniques before analysis (Rinnan, van den Berg, & Engelsen, 2009): both Savitzky–Golay and standard normal variate filters were applied to eliminate interfering spectral noise, and a first derivative filter was applied to correct for any baseline shift. All filtration techniques were carried out in SIMCA 13.0.

### 2.10 Principal component analysis

PCA was conducted on each dataset by SIMCA 13.0. Several comprehensive applications of PCA are already provided in the literature (Senior, Hamed, Masoud, & Shehata, 2012; Trufino et al., 2017). To summarize, a covariance of each matrix of dependent variables Z was determined by multiplying the matrix by its transpose. Eigenvectors and eigenvalues were determined by the software and used to deconvolute the covariance matrix. Eigenvalues were assorted in descending order and corresponding eigenvectors were multiplied by Z to get the new projected matrix as described by the first two principal components PC1 and PC2, which capture the most amount of variability in the dataset. Dataset observations were projected onto score and loadings plots by SIMCA 13.0 for analysis.

### 2.11 PLS regression

PLS regression was conducted by SIMCA 13.0 to demonstrate the dependence of mycoplasma concentration on process spectra and to further elucidate the prediction capability of the acquired process spectra on mycoplasma presence. PLS regression has previously been applied to NIR prediction methods, and these strategies are well documented (Feng, Wu, & Zeng, 2015). To summarize, loadings matrices of both spectra X and mycoplasma Y are transposed and multiplied by each corresponding score, and then added to residual matrices of variance not described by the principal components. Actual versus predicted concentrations of mycoplasma Y are plotted to assess model capabilities. Root mean square error of cross‐validation (RMSE_CV_) values are determined by SIMCA 13.0 and are used to help describe the accuracy of the model. This process was repeated for the prediction of both Day 9‐Low and Day 12‐High batch ages.

## 3 RESULTS AND DISCUSSION

### 3.1 Contamination of CHO cell bioreactor cultures with *M. arginini* results in production of mAb with increased acidic species

**Figure 1:**
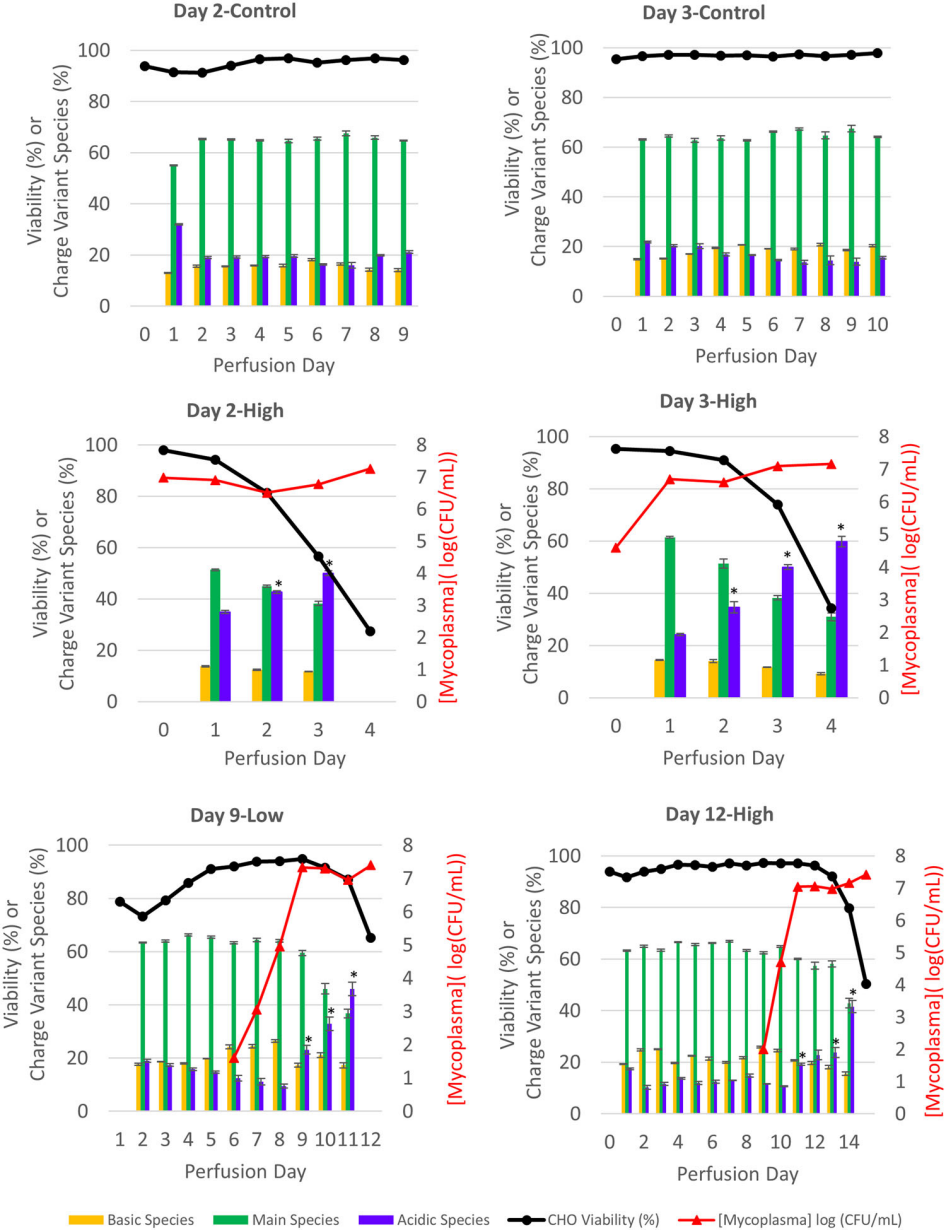
Charge distributions in mAb purified from control perfusion bioreactors and those contaminated with *Mycoplasma arginini* by perfusion day. Data represent the mean of three technical replicates and error bars represent ±1 standard deviation of the mean. Statistical significance is defined by a two‐tailed paired *t* test between corresponding control bioreactor perfusion days (Day 2‐High and Day 3‐High) or perfusion day before mycoplasma spike (Day 9‐Low and Day 12‐High) (*p < .01). For complete datasets on CHO cell growth and mycoplasma growth, please see Fratz‐Berilla et al. (2019) [Color figure can be viewed at wileyonlinelibrary.com]

During the control bioreactor runs for the models of early‐stage mycoplasma contamination events, Day 2‐Control and Day 3‐Control, CHO cell viability remained above 90% and the charge distribution of the purified mAb remained consistent throughout the runs (Figure 1). Apart from the first perfusion day of Day 2‐Control in which the percentage of main species was slightly decreased and acidic species slightly increased, the mAb purified from each day's perfusate was approximately 65% main species, 15–20% basic species, and 15–20% acidic species. During the corresponding early‐stage models of mycoplasma contamination in which *M. arginini* was spiked into the bioreactors during batch mode before the start of perfusion, Day 2‐High and Day 3‐High, acidic charge variants increased and main species decreased each day. During the late‐stage models of mycoplasma contamination in which *M. arginini* was spiked into the bioreactors during perfusion, Day 9‐Low and Day 12‐High, acidic charge variants increased significantly once *M. arginini* reached peak concentrations of 7 log(CFU/ml), which corresponds to 3 and 2 days after *M. arginini* was spiked into the bioreactors, respectively. The trend of increasing acidic species is correlated to decreasing CHO cell viabilities, which may be expected since acidic charge variants are known to increase when CHO cells are under stress (Chung et al., 2019).

### 3.2 Contamination of CHO cell bioreactor cultures with *M. arginini* reduces mAb purity

**Figure 2:**
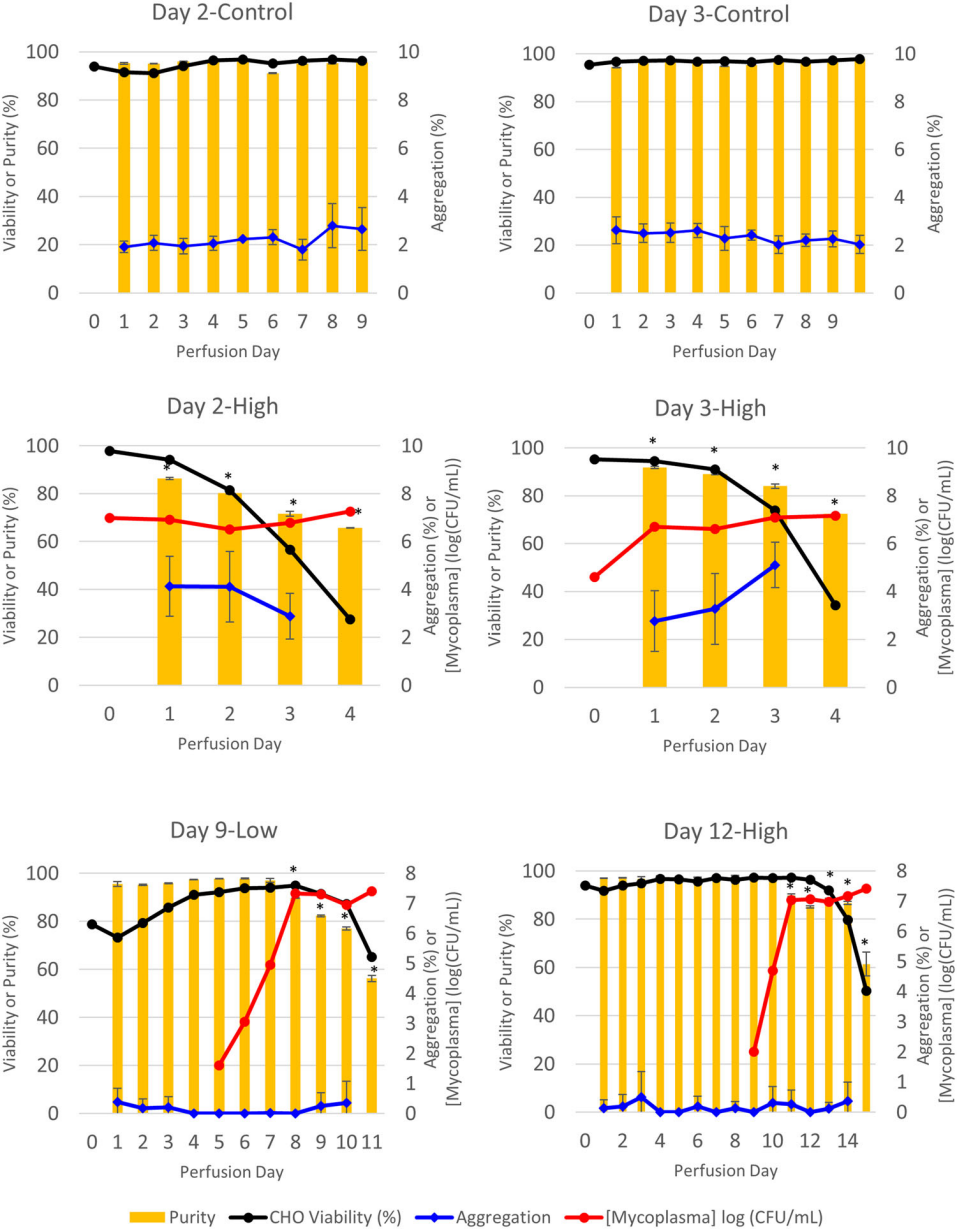
Purity and aggregation in mAb purified from control perfusion bioreactors and those contaminated with *Mycoplasma arginini* by perfusion day. Data represent the mean of three technical replicates and error bars represent ±1 standard deviation of the mean. Statistical significance is defined by two‐tailed paired *t* test between corresponding control bioreactor perfusion days (Day 2‐High and Day 3‐High) or perfusion day before mycoplasma spike (Day 9‐Low and Day 12‐High) (*p < .01) [Color figure can be viewed at wileyonlinelibrary.com]

Purity during the perfusion process, as determined by µCE‐SDS of mAb purified by Protein A chromatography, averaged 96.2 ± 1.4% during Day 2‐Control, Day 3‐Control, Day 9‐Low before *M. arginini* contamination and Day 12‐High before *M. arginini* contamination (Figure 2). Even during the initial days of perfusion of Day 9‐Low, when CHO cell viability dipped as low as 73.2%, mAb purity remained ≥95.2%. However, when *M. arginini* was introduced to the bioreactors, purity began a steady decline that was statistically significant 2–3 days after the introduction. The decreased purity was also correlated to *M. arginini* reaching peak concentrations in the bioreactor of >7 log (CFU/ml). Mab in Day 9‐Low reached the lowest measured purity of 56.3 ± 1.3%, indicating a high amount of antibody fragmentation. Furthermore, nucleic acid contamination as measured by 260:280 ratio indicated increasing amounts of nucleic acid as *M. arginini* persisted in the bioreactors (Figure S1). Noticeable increases in nucleic acid contamination (>0.55 absorbance ratio) occurred as early as a few hours after *M. arginini* was introduced to the culture. Thus, the 260:280 nm absorbance ratio of material may be a useful process parameter to monitor during downstream processing, especially in a continuous processing scheme, for the early detection of an occult contamination event.

No apparent changes were observed in the level of aggregates throughout the perfusion of CHO culture with and without mycoplasma. Because antibodies need to be purified before analysis, it is possible that the processing and purification of antibodies removed aggregates. To this point, the modest level of aggregation varied more between bioreactor runs rather than within bioreactors infected with mycoplasma. Alternatively, it is possible that aggregates are formed that are larger than the resolution of the chromatography column and are thus lost during analysis. While mass conservation was not evaluated for these samples, the recovery of the sample was not largely different. Thus, these results do not definitively show that mycoplasmas do not influence antibody aggregation; however, they do demonstrate that aggregation, depending on the downstream purification process, may not be an indicator for mycoplasma infection.

### 3.3 Contamination of CHO cell bioreactor cultures with *M. arginini* results in increased high mannose species, especially mannose 6 and higher

**Figure 3:**
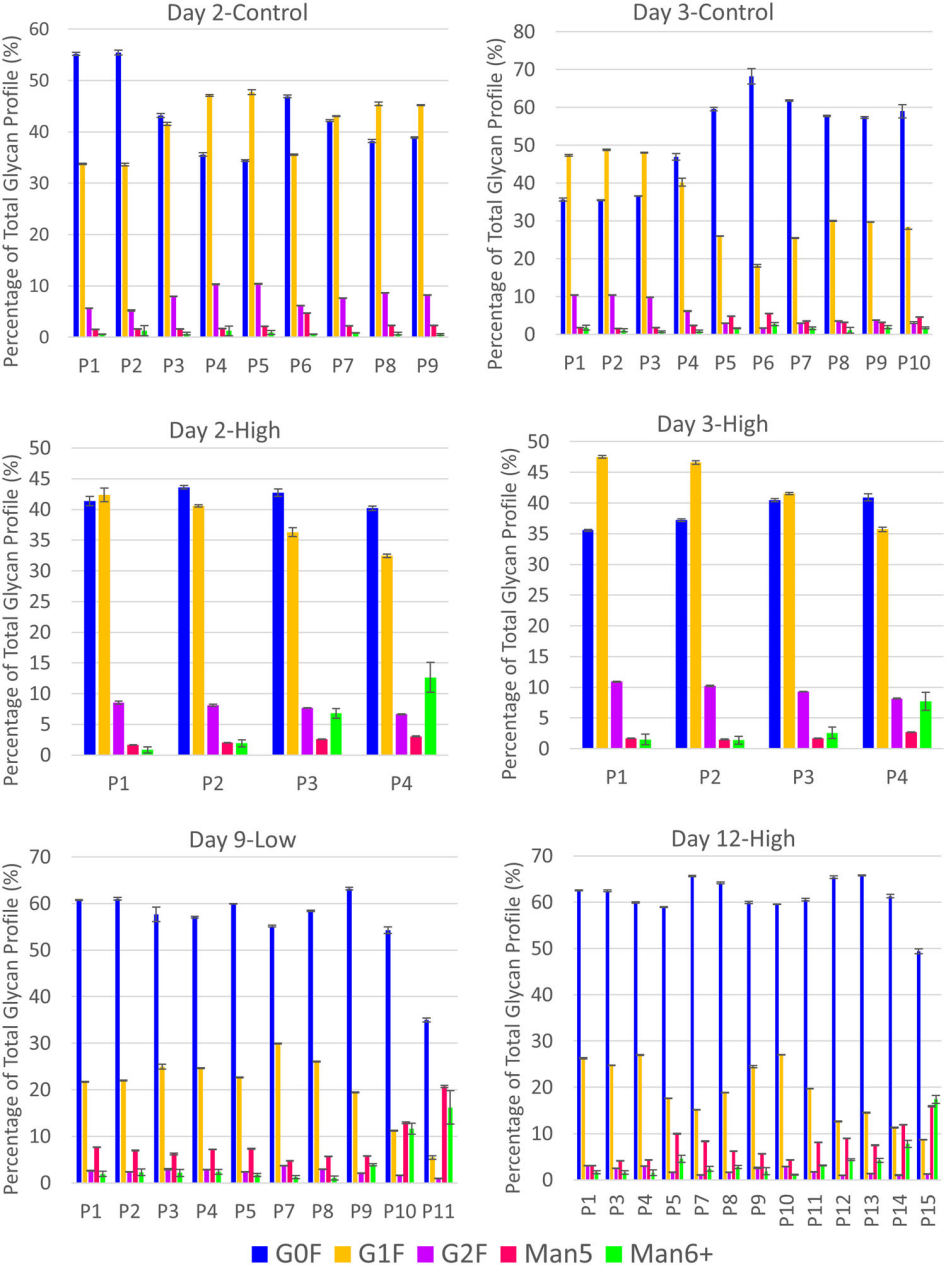
Glycan profiles in mAb purified from control perfusion bioreactors and those contaminated with *Mycoplasma arginini* by perfusion day. High mannose species, especially those with 6 mannoses or more, increase in the *M. arginini*‐contaminated bioreactors [Color figure can be viewed at wileyonlinelibrary.com]

Galactosylation varied from batch‐to‐batch and within each batch, with the largest differences being between the percentage of G0F and G1F species. G0F made up anywhere from 34% to 68% of the total glycans, and G1F varied from 15% to 49% during control operations (Figure 3). Supplementations of CHO cultures with high levels of iron and manganese are known to increase galactosylation (Crowell, Grampp, Rogers, Miller, & Scheinman, 2007; Gramer et al., 2011), and enhanced supplementation of copper and zinc have shown to have a significant impact on production and product quality as well (Graham, Bhatia, & Yoon, 2019; Graham et al., 2020; Yuk et al., 2015). However, we could not identify any overt trends between the levels of iron and manganese (Figures S1 and S2) and the amount of galactosylation. The high variability between and within batches makes it difficult to interpret the potential causes of change in galactosylation profiles.

**Figure 4:**
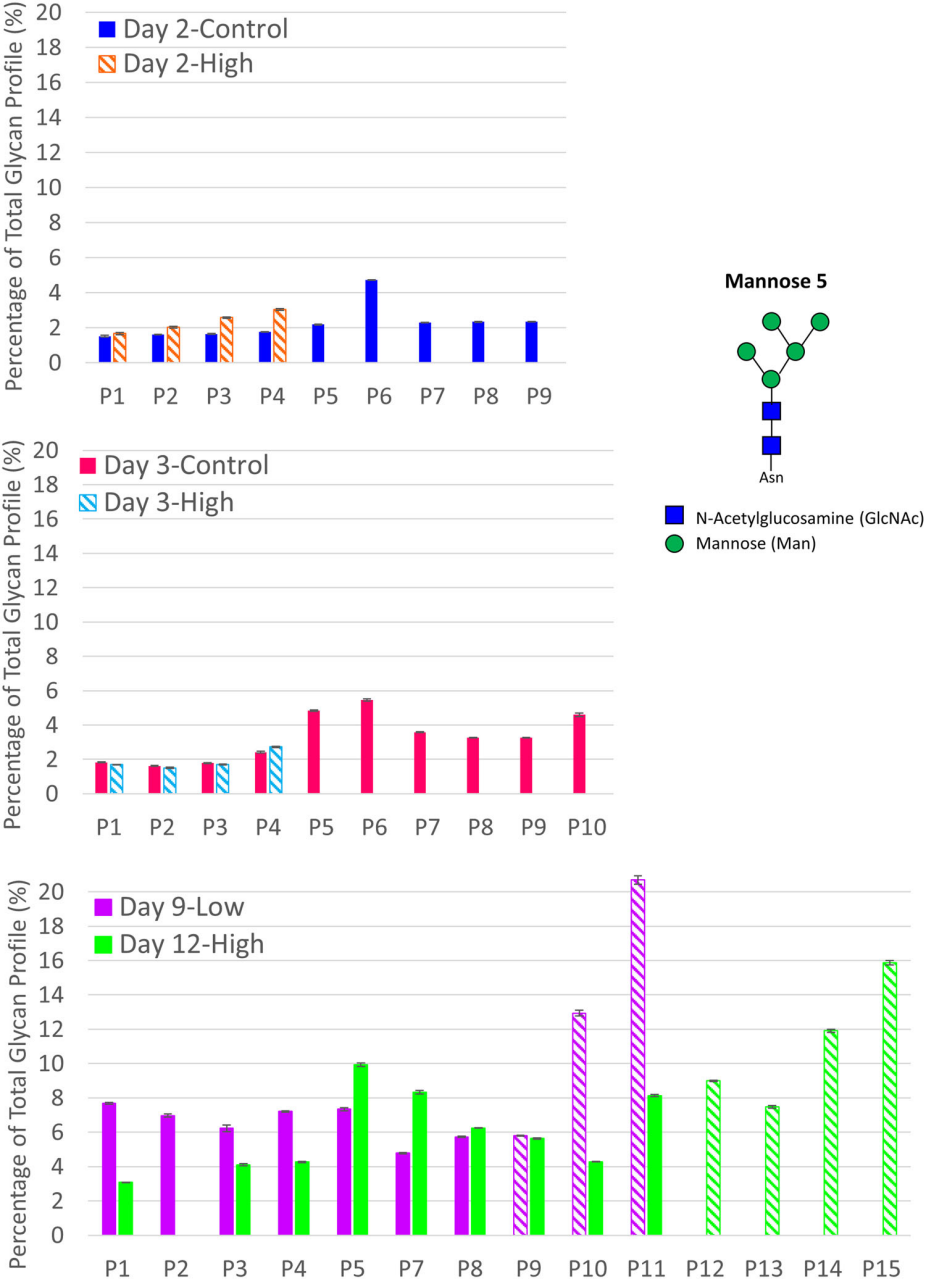
Mannose 5 species in mAb purified from control perfusion bioreactors and those contaminated with *Mycoplasma arginini* by perfusion day. Solid bars indicate perfusion days in which the bioreactor is uncontaminated and striped bars indicate the presence of contamination. The top panel compares Day 2‐High and Day 2‐Control, the middle panel compares Day 3‐High and Day 3‐Control, and the bottom panel compares Day 9‐Low and Day 12‐High [Color figure can be viewed at wileyonlinelibrary.com]

**Figure 5:**
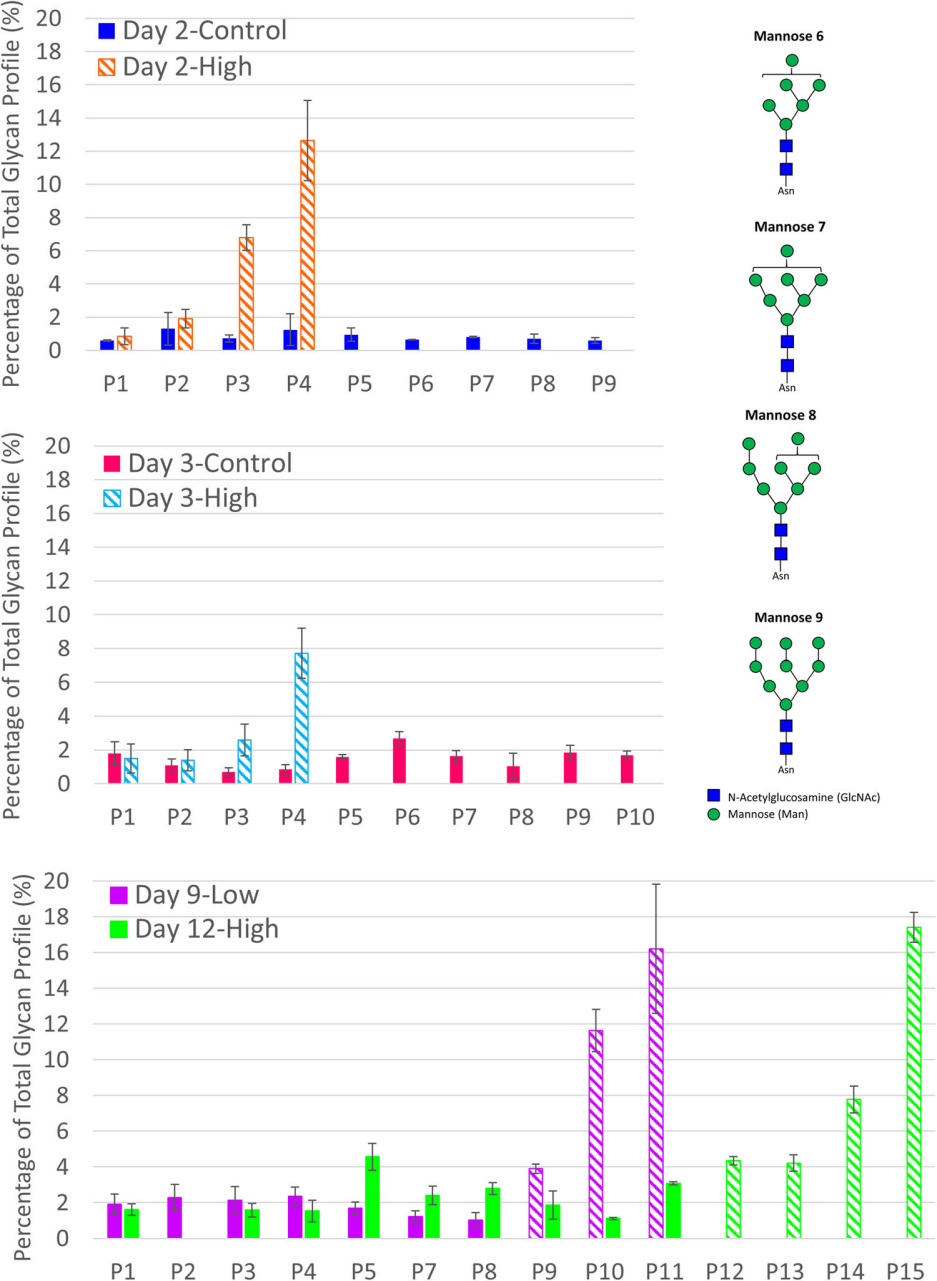
Mannose 6–9 species in mAb purified from control perfusion bioreactors and those contaminated with *Mycoplasma arginini* by perfusion day. Solid bars indicate perfusion days in which the bioreactor is uncontaminated and striped bars indicate the presence of contamination. The top panel compares Day 2‐High and Day 2‐Control, the middle panel compares Day 3‐High and Day 3‐Control, and the bottom panel compares Day 9‐Low and Day 12‐High [Color figure can be viewed at wileyonlinelibrary.com]

The presence of high mannose species have been associated with periods of CHO cell stress (Fan et al., 2015; Powers et al., 2019; Villacres, Tayi, Lattova, Perreault, & Butler, 2015), increased specific productivity (Zalai et al., 2016), and high concentrations of manganese and limited glucose (Surve & Gadgil, 2015), and thus we expected that high mannose species would increase in the presence of *M. arginini*. A noticeable and significant trend in glycan profile exists between *M. arginini* presence and high mannose species, particularly for those with 6 or more mannose residues (Figures 4 and 5). Interestingly, levels of mannose 5 species did not apparently increase in Day 2‐High and Day 3‐High (Figure 4), but combined levels of mannose 6–8 species trend higher in contrast to the same perfusion days of Day 2‐Control and Day 3‐Control, respectively (Figure 5). For Day 9‐Low and Day 12‐High, some increases in mannose 5 species are apparent (Figure 4). Increases in levels of mannose 6–9 species reach 16.2% and 17.4% of the total glycan profile for Day 9‐Low and Day 12‐High, respectively, by the final day of perfusion (Figure 5). These high mannose species containing higher than 5 mannose residues are produced early in the oligosaccharide modification scheme that occurs in the Golgi, indicating that mycoplasma contamination is causing significant enough intracellular disturbances to disrupt the glycan reaction pathways at the beginning stages.

### 3.4 PCA indicates that mycoplasma concentration is positively correlated with high mannose species and acidic charge variants

**Figure 6:**
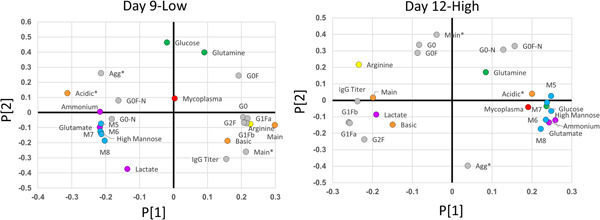
PCA loadings plots of Day 9‐Low and Day 12‐High. Culture data included are concentration of mycoplasma (red), IgG titer (gray), nutrients (glucose and glutamine—green), arginine (yellow), and waste (ammonium, lactate, and glutamate—purple). Product quality data included are glycan species (G0F‐N, G0F, G0‐N, G0, G1Fa, G1Fb—gray; M5, M6, M7, M8, high mannose—blue), aggregate and nonaggregate (main) species (gray; * distinguishes aggregate data) and charge variant species (orange). IgG, immunoglobulin G; PCA, principal component analysis [Color figure can be viewed at wileyonlinelibrary.com]

PCA was performed to assess the suitability of using multivariate data analysis (MVDA) to characterize the impact of mycoplasma presence on metabolites and critical quality attributes. Only Day 12‐High and Day 9‐Low were fully investigated because they had a sufficient number of perfusate days to obtain mAb and thus collect CQA data. In Day 12‐High, mycoplasma presence has a positive correlation with a cluster of M5, M6, M7, and other high mannose species along the first principal component. Glutamate and ammonium concentrations also positively correlate with this cluster along with glucose concentration and acidic charge variants (Figure 6). However, glucose concentration and acidic charge variants also show a slightly negative (but less influential) correlation along the second principal component. A negatively correlated cluster of G1F and G2F glycan species, IgG titer, lactate concentration, and basic and main charge variants also exist along the first principal component. The polarity of these two clusters demonstrates the significant influence of both on the component. Furthermore, the degree of separation between the two reveals both impactful negative and positive correlation between these key processes and quality parameters.

The loadings plot of Day 9‐Low similarly depicts that the two polarized clusters negatively correlated along the first principal component (Figure 6). Both G0‐N and G0F‐N species also show a positive correlation with the high mannose cluster. However, mycoplasma concentration falls just outside the cluster toward the midpoint of the first principal component. Although mycoplasma has a weaker influence on the first component in comparison to Day 12‐High, the Day 9‐low loadings plot indicates a slightly positive correlation between mycoplasma and high mannose glycan species and acidic charge variants.

The relatedness of parameters in the positive cluster of the first principal component of both Day 12‐High and Day 9‐Low falls in accordance with the literature, as high mannose species have shown to yield acidic antibody variants (Du et al., 2012). Moreover, the presence of ammonia accumulation has long been known to be a byproduct of some mycoplasma presence via arginine depletion (Matsuura, Seto, & Watanabe, 1990). Although less influential in Day 9‐Low, mycoplasma still has a positive correlation with the positive cluster. The cluster along the negative end of the first principal component contains glycans and charge variants more favorable to CHO cell culture, as G1F and G2F glycan species have a positive correlation with IgG titer and main charge variants. These relationships underline the link between mycoplasma contamination and the more dubious CHO cell culture artifacts.

### 3.5 NIR spectra PLS model indicates low and high extreme mycoplasma presence

**Figure 7:**
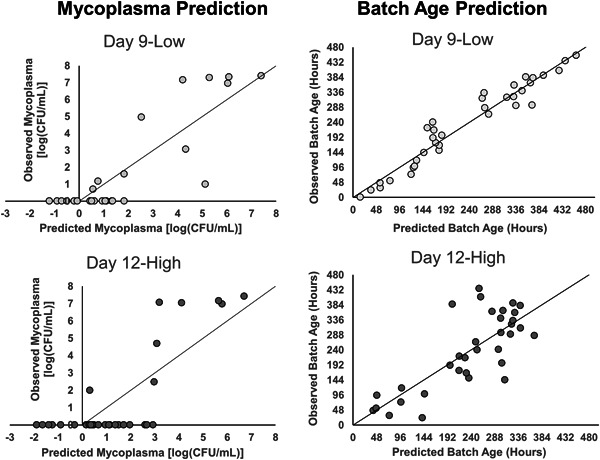
PLS models of NIR spectra to predicted mycoplasma contamination in Day 9‐Low and Day 12‐High. NIR spectra can predict mycoplasma contaminations at high (4–8 log [CFU/ml]) concentrations, but some false positives occur at low mycoplasma concentrations. The batch age predictions indicate a strong linear correlation and lack of polarized clustering between the predicted and observed batch age, which provides evidence of reliability of the saturated mycoplasma clusters in the prediction models. CFU, colony‐forming unit; NIR, near‐infrared; PLS, partial least square

PLS models were constructed in SIMCA 13.0 and principal components were added until greater than 90% of variability was captured by each model (Table 2). In both Day 12‐High and Day 9‐Low models (Figure 7), a cluster of points of no mycoplasma presence is within the predicted mycoplasma range from −2 to 2 log (CFU/ml). Moreover, both models predict saturated mycoplasma concentrations at a range of approximately 4 to 7.5 log (CFU/ml). To explore the effects of batch age on the mycoplasma models, separate batch age prediction models were constructed using the same NIR spectra. The more evident linearity and lack of polarized clustering on the batch age plots provide insight into the reliability of the saturated mycoplasma clusters in the prediction models.

**Table 2:** PCA and PLS model statistics of Day 12‐High and Day 9‐Low

	PCA			PLS			
	A	N	*R* ^2^ *X*	A	*R* ^2^ *X*	*Q* ^2^ *X*	RMSE_CV_
Day 12‐High	10	29	.988	4	.936	0.347	2.147
Day 9‐Low	8	26	.989	3	.971	0.485	2.027

*Note*: A, principal components; N, observations.

Abbreviations: PCA, principal component analysis; PLS, partial least square; RMSE_CV_, root mean square error of cross‐validation.

Accurate prediction of mycoplasma concentrations by these two NIR models lacks feasibility based on *Q* ^2^ prediction and RMSEV values (Table 2). However, the two clusters representing both no mycoplasma presence and mycoplasma saturation do provide an avenue to utilize the NIR prediction models for low and high alarm signaling. As both models dependably reveal the presence of saturated mycoplasma after a prediction >4 log (CFU/ml), application of these models for mycoplasma saturation detection is possible.

## 4 CONCLUSION

In this study, we furthered our examination of the impacts of a mycoplasma contamination event on a model CHO cell bioprocess to include the detrimental effects of *M. arginini* presence on the CQAs of a model IgG1. Previously, we showed that *M. arginini* grew and persisted in a serum‐free bioreactor culture of a CHO DG44 cell line expressing a model monoclonal IgG1 antibody (Fratz‐Berilla et al., 2019). Here, we investigated the effects of mycoplasma presence on IgG CQAs and found that contamination increased acidic charge variants, increased DNA contamination, and led to very high percentages of specific high mannose species: those with 6 or more mannose residues. We used MVDA to more thoroughly dissect and find relationships among our complex datasets containing information on CHO cell growth, mycoplasma growth, medium nutrients and waste, trace metals, CQAs, and NIR spectra. PCA allowed us to confirm some biologically relevant relationships but the considerable batch‐to‐batch variability diluted what may be more subtle correlations that would not be found using only univariate analysis on the individual factors. However, using PCA, we observed that mycoplasma presence did have a positive correlation with acidic charge variants and high mannose species along the first principal component, but the correlations were much stronger with Day 12‐High than in Day 9‐Low, where mycoplasma had a less significant impact along the first principal component, possibly indicating that the lower mycoplasma spike concentration may have influenced the culture dynamics more than could be previously deciphered by univariate analysis, or that the complexity of the culture was not fully captured in the datasets collected. Using PLS, we found that NIR spectra predicted mycoplasma contaminations at high (4–8 log [CFU/ml]) concentrations, but some false positives occur at low mycoplasma concentrations. With the growing desire to use spectral data, such as Raman and NIR, along with MVDA and modeling to monitor and control upstream bioprocesses, it may be important to understand what a contamination event, especially an otherwise occult contamination event, may have on bioprocess spectral data. The PLS model shown here may provide an avenue to utilize the NIR prediction models for low and high alarm signaling, but as it exists at present would not likely be efficient as an early indicator of *an M. arginini* contamination event.

## CONFLICT OF INTERESTS

The authors declare that there are no conflict of interests.

## Supporting information

Supporting information

Supporting information

Supporting information

Supporting information
