# Supplementary figures and images for: Impacts on product quality attributes of monoclonal antibodies produced in CHO cell bioreactor cultures during intentional mycoplasma contamination events
Source: Biotechnol Bioeng. 2020 Jun 4;117(9):2802–15. doi: 10.1002/bit.27436 (PMC7496122; doi:10.1002/bit.27436)

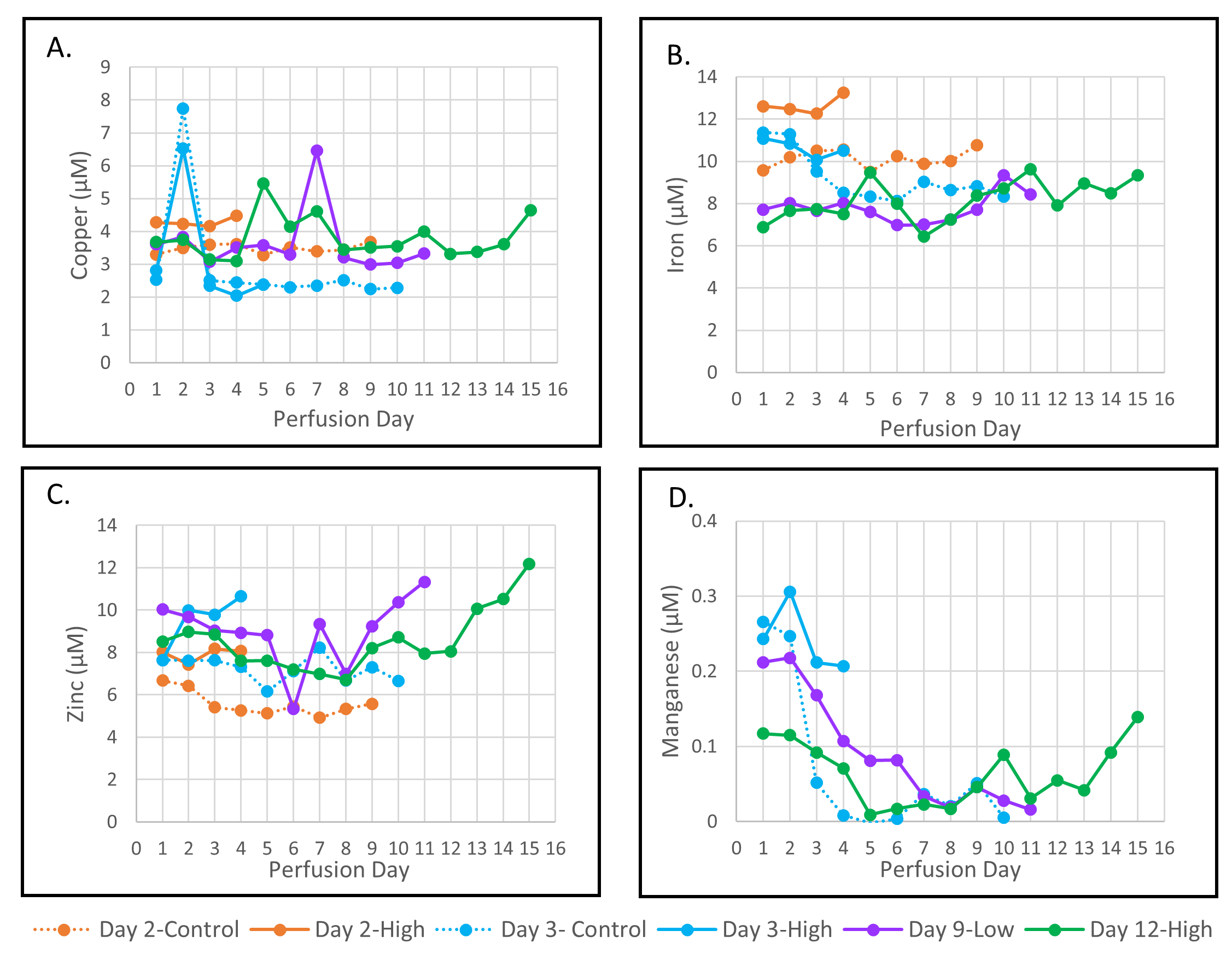

Supplement: Supplementary file 1 — Supporting information [file BIT-117-2802-s001.tif]

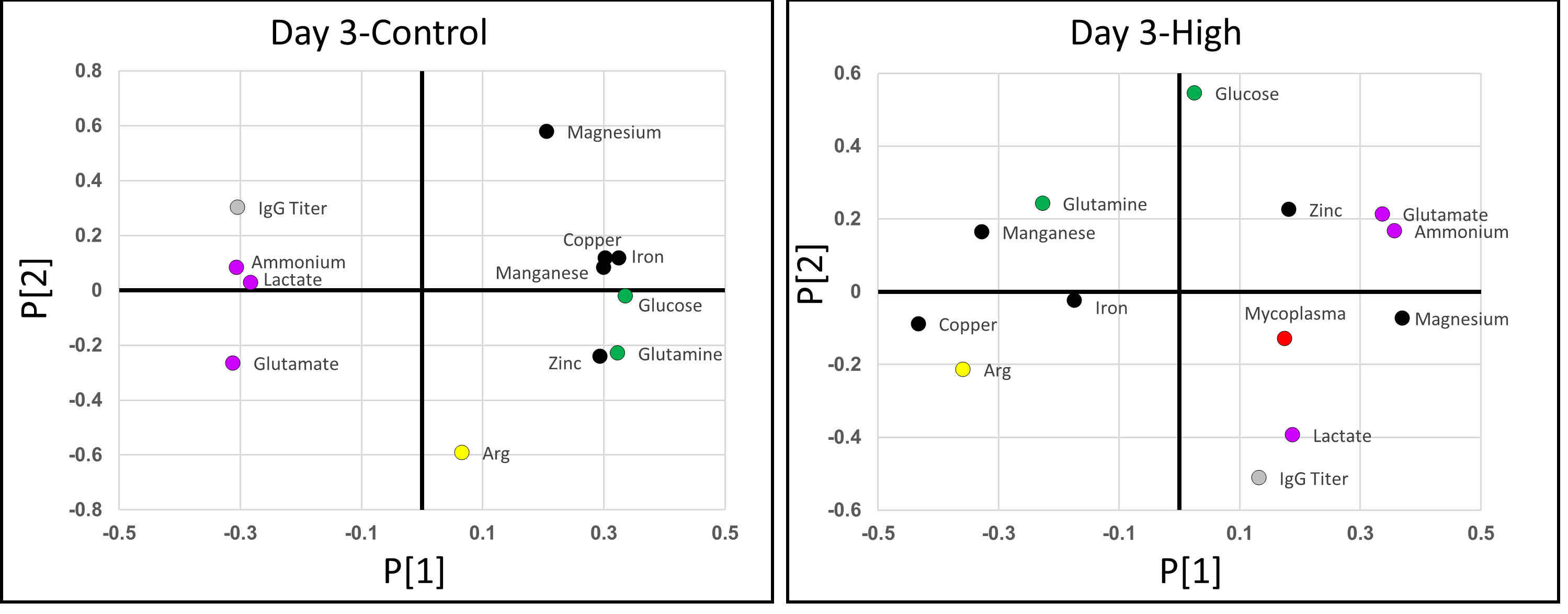

Supplement: Supplementary file 2 — Supporting information [file BIT-117-2802-s002.tif]

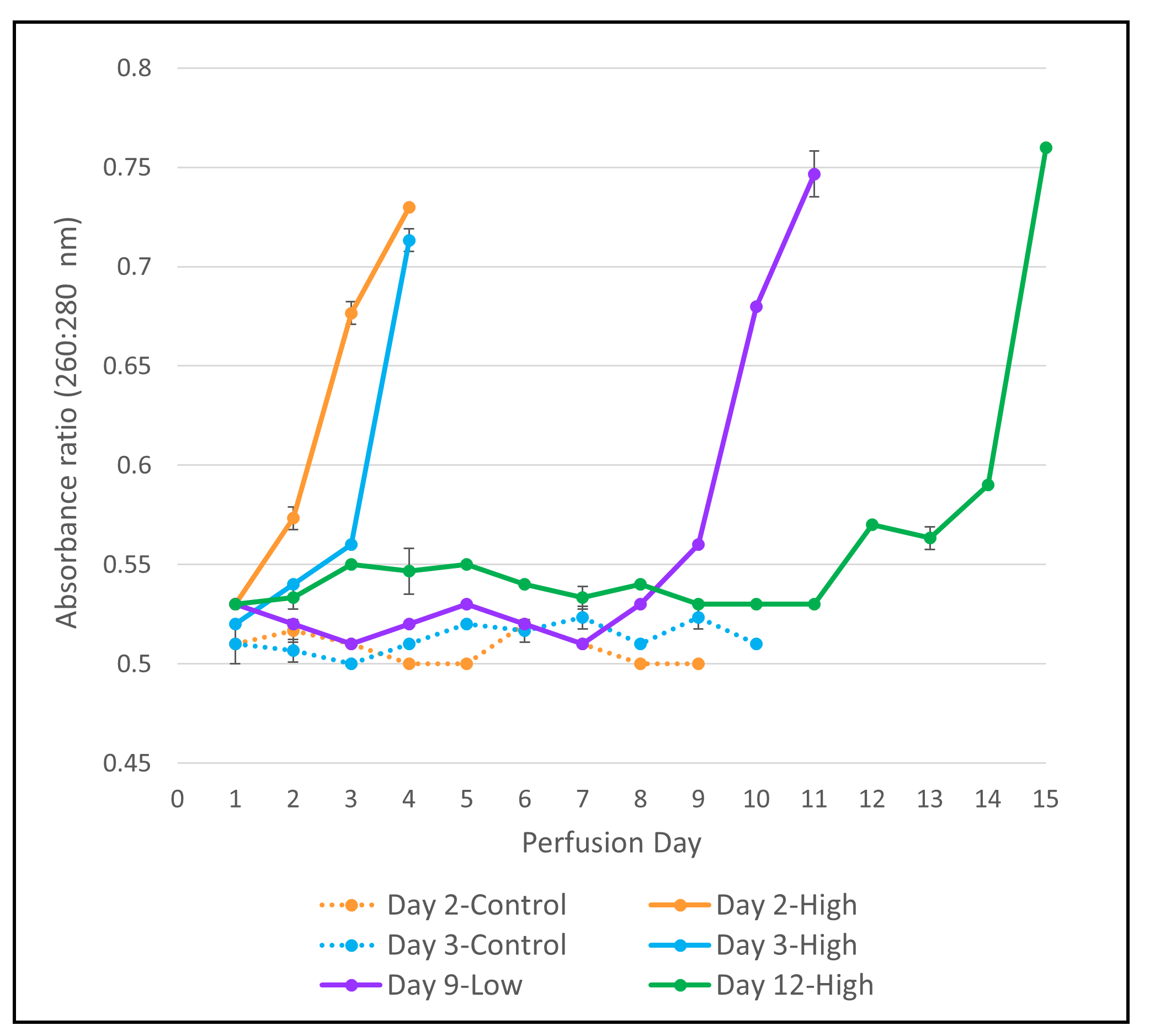

Supplement: Supplementary file 3 — Supporting information [file BIT-117-2802-s003.tif]
