# Supplementary material for: Impacts on product quality attributes of monoclonal antibodies produced in CHO cell bioreactor cultures during intentional mycoplasma contamination events
Source: Biotechnol Bioeng. 2020 Jun 4;117(9):2802–15. doi: 10.1002/bit.27436 (PMC7496122; doi:10.1002/bit.27436)
Supplement: Supplementary file 4 — Supporting information [file BIT-117-2802-s004.docx]

**Supplementary Figure 1. Copper, iron, zinc, and manganese levels in control perfusion bioreactors and those contaminated with *M. arginini.*** Copper (A.), iron (B.), and zinc (C.) remained at similar levels throughout the run, but manganese, which is at sub-micromolar concentration in the basal media, (D.) decreases in the high cell density cultures.

**Supplementary Figure 2. PCA loadings plots of Day 3-Control and Day 3-High.** Culture data included are concentration of mycoplasma (red), IgG titer (gray), nutrients (glucose and glutamine—green), arginine (yellow), waste (ammonium, lactate, and glutamate—purple), and metals (copper, iron, zinc, manganese, and magnesium—black).

**Supplementary Figure 3. 260:280 nm absorbance ratios in purified samples from control perfusion bioreactors and those contaminated with *M. arginini.*** Ratios of 0.57 and below indicate little or no nucleic acid impurities. During perfusion days when *M. arginini* concentration peaks, nucleic acid contaminants increase.
